# Association of peptic ulcer disease with obesity, nutritional components, and blood parameters in the Korean population

**DOI:** 10.1371/journal.pone.0183777

**Published:** 2017-08-24

**Authors:** Jihye Kim, Keun Ho Kim, Bum Ju Lee

**Affiliations:** KM Fundamental Research Division, Korea Institute of Oriental Medicine, Daejeon, Republic of Korea; University Hospital Llandough, UNITED KINGDOM

## Abstract

**Objectives:**

Peptic ulcer disease (PUD) is a common disorder, but whether an association exists between PUD and anthropometric indicators remains controversial. Furthermore, no studies on the association of PUD with anthropometric indices, blood parameters, and nutritional components have been reported. The aim of this study was to assess associations of anthropometrics, blood parameters, nutritional components, and lifestyle factors with PUD in the Korean population.

**Methods:**

Data were collected from a nationally representative sample of the South Korean population using the Korea National Health and Nutrition Examination Survey. Logistic regression was used to examine associations of anthropometrics, blood parameters and nutritional components among patients with PUD.

**Results:**

Age was the factor most strongly associated with PUD in women (p = <0.0001, odds ratio (OR) = 0.770 [0.683–0.869]) and men (p = <0.0001, OR = 0.715 [0.616–0.831]). In both crude and adjusted analyses, PUD was highly associated with weight (adjusted p = 0.0008, adjusted OR = 1.251 [95%CI: 1.098–1.426]), hip circumference (adjusted p = 0.005, adjusted OR = 1.198 [1.056–1.360]), and body mass index (adjusted p = 0.0001, adjusted OR = 1.303 [1.139–1.490]) in women and hip circumference (adjusted p = 0.0199, adjusted OR = 1.217 [1.031–1.435]) in men. PUD was significantly associated with intake of fiber (adjusted p = 0.0386, adjusted OR = 1.157 [1.008–1.328], vitamin B2 (adjusted p = 0.0477, adjusted OR = 1.155 [1.001–1.333]), sodium (adjusted p = 0.0154, adjusted OR = 1.191 [1.034–1.372]), calcium (adjusted p = 0.0079, adjusted OR = 1.243 [1.059–1.459]), and ash (adjusted p = 0.0468, adjusted OR = 1.152 [1.002–1.325] in women but not in men. None of the assessed blood parameters were associated with PUD in women, and only triglyceride level was associated with PUD in men (adjusted p = 0.0169, adjusted OR = 1.227 [1.037–1.451]).

**Discussion:**

We found that obesity was associated with PUD in the Korean population; additionally, the association between nutritional components and PUD was greater in women than in men.

## Introduction

Peptic ulcer disease (PUD) is a common and serious digestive system disease that includes gastric and duodenal ulcers [1, 2]. The prevalence of PUD is approximately 4.1%, and approximately 10% of people develop PUD in their lifetime [1, 3]. In the United States (US), approximately 15–18 million people have PUD [3, 4]. According to a study published by the Health Insurance Review and Assessment Service in 2010, approximately 4 million patients had PUD each year from 2005–2008 [5].

Previous studies have reported that *Helicobacter pylori (H*. *pylori)* infection increases the risk of PUD. Infection with *H*. *pylori* is one of the major etiologies of PUD and is considered a common pathogenic mechanism that drives the development of PUD [6–9]. Although *H*. *pylori* is the most important risk factor of PUD, 5–20% of PUD patients exhibit no evident cause [10]. Recent studies have shown that PUD is strongly associated with non-organic factors such as lifestyle, psychological stress, obesity, and intake, and these non-organic causes may play a role in the onset and course of PUD [9, 11–15].

Many studies to date have assessed the association of PUD with anthropometric indices, nutritional components, and lifestyle. Obesity and visceral obesity have been related to disruptions in normal epithelial barrier function and to systemic inflammation [14, 16]. However, although several studies have reported an association of PUD with anthropometric indices such as body mass index (BMI), waist-to-height ratio (WHtR), and waist circumference (WC), the association between PUD and obesity remains unclear [2, 15–19]. Several studies have reported a relationship between PUD and anthropometric indices including BMI, WHtR, and WC [15–17], while others have suggested that PUD is not associated with BMI and/or WHtR [2, 18, 19].

In the nutrition, several previous studies have reported that the intake of particular nutritional components is associated with PUD and gastrointestinal diseases [20–25]. High fiber intake has been related to a low prevalence of PUD and gastroesophageal reflux disease [20, 21]. Vitamin A may prevent duodenal ulcer [21, 22], and vitamin C plays a key protective role in PUD and its complications [25].

Furthermore, several studies have shown that aging, smoking, and sleep duration may be risk factors for PUD. The prevalence of PUD increases in both men and women as they grow older [3, 26]. Smoking increases the risk of PUD in men and women and in all age groups, and the risk of PUD is higher among non-smoking men than non-smoking women [27]. Additionally, people who sleep more than 9 hours are less likely to develop PUD than those who sleep less than 9 hours [28].

Although many studies have been conducted to clarify the risk factors of PUD, the association of PUD with obesity has been controversial, and the associations that exist between PUD and various risk factors such as lifestyle, blood parameters, and nutrition remains to be fully understood. The objective of this study was to examine the association of PUD with anthropometric indices, nutritional components, blood parameters, and lifestyle factors in the Korean population. The results of this study will provide basic information for clinical studies on patients with PUD and evidence for the prevention and management of PUD patients. To the best of our knowledge, this is the first study to explore the association of PUD with anthropometrics, blood parameters, lifestyle, and nutrition in the Korean population.

## Material and methods

### Data source

This study was based on data obtained from the First Korea National Health and Nutrition Examination Survey (KNHANES I), which was a prospective cross-sectional nationally representative survey study conducted by the Korea Centers for Disease Control and Prevention (KCDC) in 1998 to estimate the general health and nutritional status of the Korean population [29]. Specifically, KNHANES I through VI were conducted from 1998 to 2014. KNHANES I was conducted in 1998 as a triennial survey. KNHANES II-VI were conducted in 2001 (KNHANES II), 2005 (KNHANES III), 2007–2009 (KNHANES IV), 2010–2012 (KNHANES V), and 2013–2015 (KNHANES VI), respectively [30, 31]. KNHANCES I (1998) defined PUD as including gastritis, gastric ulcers, and duodenal ulcers [29]. In contrast, KNHANES II-IV (2001–2009) defined PUD as including only gastric and duodenal ulcers, except for gastritis [32]. KNHANES V and IV (2010–2015) did not include the diagnostic results for PUD [33]. Therefore, it was difficult to integrate the data from KNHANCES I and KNHANES II-VI because the PUD patients were recruited based on different PUD diagnosis criteria in each survey. Although KNHANES II-VI may derive more valuable findings based on a larger and more representative data set on PUD compared with the findings from KNHANCES I, we chose KNHANCES I for this PUD study because we focused on a wider range of PUD criteria, including gastritis, gastric ulcer, and duodenal ulcer, and many epidemiological and medical studies have revealed a very strong association of gastritis with duodenal and gastric ulcers [34–36]. Therefore, we conducted an observational study on the association between a wider range of PUD, including gastritis, and various parameters such as anthropometric indices, blood parameters, lifestyles, and nutritional components.

The KNHANES I was approved by the Korean Ministry of Health and Welfare and was conducted according to the Declaration of Helsinki [29, 37]. The KNHANES I adopted a stratified and multi-stage clustered probability sampling method to select a representative sample of the non-institutionalized Korean population. To use open source data from the KNHANES I, this study was approved by the Institutional Review Board of the Korea Institute of Oriental Medicine with a waiver of documentation of informed consent (IRB No. I-1703/002-004).

### Participants and definition

The 1998 KNHANES I included 39,060 subjects in 15 cities (Seoul, Busan, Daegu, Incheon, Gwangju, Daejeon, Gyeonggi-do, Gangwon-do, Chungcheongbuk-do, Chungcheongnam-do, Jeollabuk-do, Jeollanam-do, Gyeongsangbuk-do, Gyeongsangnam-do and Jeju island). Only participants older than 30 years of age were included in this study. PUD was diagnosed by a doctor and included gastritis, gastric ulcer, and duodenal ulcer. Subjects with missing data were excluded, resulting in a final sample of 4,652 participants (2,126 male and 2,526 female) ranging in age from 30–70 years. The Supporting Information explains the sample selection procedure (S1 Fig).

### Measurements

The data used in this study were derived from the following: health-related interviews, health examinations, and nutrition surveys. The associations between anthropometric indices and various diseases have been frequently examined [38–41]. In this study, we selected six commonly used indices including weight, height, BMI, WHtR, WC, and hip circumference (HipC), based on previous studies, to examine the association between PUD and anthropometric indices [38–41]. Additionally, the associations between PUD and seven blood parameters including triglyceride and glucose levels and sixteen nutritional components including fiber, potassium, and vitamins were analyzed. The demographic and baseline characteristics assessed in this study are listed in Table 1.

**Table 1:** Demographic characteristics of all study variables.

Index	Male		Female	
	Control	Peptic ulcer	Control	Peptic ulcer
Numbers	1944	182	2232	294
Age (mean, SD)	46.30 (11.31)	50.20 (11.02)	46.65 (11.62)	49.73 (1.240)
Education (No. of subjects, %)				
Uneducated	67 (3.4)	9 (4.9)	307 (13.7)	60 (20.4)
Elementary school	364 (18.7)	46 (25.3)	590 (26.4)	99 (33.7)
Middle school	326 (16.8)	39 (21.4)	411 (18.4)	51 (17.3)
High school	742 (38.2)	62 (34.1)	672 (30.1)	63 (21.4)
University	445 (22.9)	26 (14.3)	252 (11.3)	21 (7.1)
Occupation (No. of subjects, %)				
White-collar worker	186 (9.6)	11 (6.0)	83 (3.7)	5 (1.7)
Office worker	225 (11.6)	18 (9.9)	65 (2.9)	6 (2.0)
Service	341 (17.5)	23 (12.6)	371 (16.6)	43 (14.6)
Farmer and fisher	379 (19.5)	51 (28.0)	410 (18.4)	67 (22.8)
Blue-collar worker	551 (28.3)	51 (28.0)	259 (11.6)	39 (13.3)
Soldier	1 (0.1)	-	-	-
Student	2 (0.1)	-	3 (0.1)	-
Housewife	2 (0.1)	-	875 (39.2)	104 (35.4)
Unemployed	257 (13.2)	28 (15.4)	166 (7.4)	30 (10.2)
Smoking status (No. of subjects, %)				
Current smoker	1208 (62.1)	112 (61.5)	107 (4.8)	19 (6.5)
Smokes occasionally	67 (3.4)	6 (3.3)	17 (0.8)	2 (0.7)
Ex-smoker	342 (17.6)	42 (23.1)	37 (1.7)	6 (2.0)
Never smoker	327 (16.8)	22 (12.1)	2071 (92.8)	267 (90.8)
Alcohol consumption (No. of subjects, %)				
Frequently drink	674 (34.7)	64 (35.2)	77 (3.4)	10 (3.4)
Occasionally drink	660 (64.0)	53 (29.1)	517 (23.2)	55 (18.7)
Rarely drink	248 (12.8)	23 (12.6)	511 (22.9)	68 (23.1)
Ex-drinker	149 (7.7)	19 (10.4)	66 (3.0)	10 (3.4)
Never drinker	213 (11.0)	23 (12.6)	1061 (47.5)	151 (51.4)
Sleep duration (mean, SD)^‡^	6.92 (1.24)	6.93 (1.22)	6.56 (0.61)	6.75 (1.31)
*Vital signs*				
Pulse rate per 15 sec	18.21 (2.560)	17.92 (2.270)	18.27 (2.350)	18.30 (20.18)
Systolic BP (mmHg)^‡^	128.9 (19.06)	126.2 (19.05)	124.9 (20.18)	123.4 (11.29)
Diastolic BP (mmHg)^‡^	82.52 (12.24)	80.32 (11.54)	78.02 (11.63)	76.29 (19.82)
*Anthropometrics*				
Weight (kg)^‡^	66.37 (9.700)	63.99 (9.340)	57.55 (8.290)	55.70 (5.610)
Height (cm)^‡^	168.1 (6.060)	167.3 (5.860)	155.7 (5.710)	155.0 (8.680)
Body mass index (kg/m^2^)^‡^	23.44 (2.890)	22.85 (3.020)	23.75 (3.230)	23.17 (0.060)
Waist-to-height ratio^‡^	0.500 (0.050)	0.500 (0.050)	0.510 (0.060)	0.510 (6.000)
Waist circumference (cm)^‡^	83.97 (7.950)	82.82 (8.620)	79.84 (9.210)	78.86 (3.030)
Hip circumference (cm)	93.83 (6.200)	92.09 (6.130)	94.12 (6.760)	92.93 (1.300)
*Blood parameters*				
Hemoglobin (g/dl)^‡^	15.26 (1.230)	15.13 (1.260)	13.08 (1.250)	13.03 (3.640)
Platelet (%)^‡^	23.44 (5.430)	23.43 (5.600)	24.49 (5.450)	23.84 (9.980)
Cholesterol (mg/dl)	190.5 (37.54)	189.5 (31.37)	191.3 (37.02)	191.3 (59.13)
Triglyceride (mg/dl)^‡^	143.0 (67.63)	130.8 (58.56)	114.8 (56.18)	117.7 (12.43)
HDL cholesterol (mg/dl)^‡^	47.60 (12.69)	47.50 (10.41)	50.99 (12.26)	50.67 (33.84)
Glucose (mg/dl)	103.6 (31.41)	99.90 (24.97)	101.6 (35.35)	102.0 (5.600)
Hemoglobin A1c (HbA1c, %)	5.570 (5.750)	5.170 (0.710)	5.640 (7.000)	5.420 (3.620)
*Dietary intake per day*				
Energy (kcal)^‡^	2204 (839.6)	2086 (858.4)	1780 (713.1)	1687 (428.8)
Water (g)^‡^	938.5 (536.1)	865.2 (464.9)	784.9 (468.0)	727.0 (89.59)
Carbohydrate (g)^‡^	351.9 (130.7)	340.5 (119.1)	310.5 (123.2)	294.5 (3.800)
Fiber (g)^‡^	7.960 (4.320)	7.420 (4.140)	7.030 (4.090)	6.350 (9.350)
Ash (g)^‡^	22.65 (12.08)	20.81 (11.28)	18.26 (10.45)	16.33 (252.2)
Calcium (mg)^‡^	553.6 (432.1)	495.6 (356.4)	476.4 (355.3)	402.8 (486.2)
Phosphorus (mg)^‡^	1228 (552.6)	1119 (523.1)	988.3 (479.0)	929.7 (7.940)
Iron (mg)^‡^	14.97 (9.450)	14.08 (8.220)	12.42 (8.590)	11.35 (2473)
Sodium (mg)^‡^	5834 (3585)	5220 (3207)	4596 (2936)	4052 (1271)
Potassium (mg)^‡^	2951 (1470)	2781 (1489)	2536 (1459)	2280 (489.8)
Vitamin A (μgRE)^‡^	717.4 (863.8)	690.2 (923.7)	583.3 (704.9)	481.2 (2814)
Vitamin B2 (Riboflavin, mg)^‡^	1.160 (0.750)	1.080 (0.820)	0.950 (0.710)	0.800 (8.900)
Vitamin C (mg)	129.7 (105.3)	128.5 (103.6)	136.9 (125.2)	118.5 (0.000)
Carotene (μg)^‡^	3726 (4675)	3791 (5477)	3064 (3777)	2576 (71.35)
Retinol (μg)^†^	78.57 (224.8)	48.76 (82.94)	61.11 (270.7)	43.49 (0.720)
Niacin (μg)^‡^	19.25 (12.09)	17.32 (10.36)	14.87 (9.700)	13.85 (99.81)

Data were collected from the Korea National Health and Nutrition Examination Survey (1998), Republic of Korea. The data are represented as the mean ± standard deviation or as numbers of participants and percentages, N (%), for continuous and categorical variables, respectively.

^†^ p <0.05 and ‡ p <0.01 between men and women using an independent two-sample t-test.

### Statistical analysis

Statistical analyses were performed using SPSS 19 for Windows (SPSS Inc., Chicago, IL, USA). In the crude analysis and the analyses adjusted for age, education, job, smoking, and drinking, binary logistic regression was conducted to identify significant differences between the PUD group (0) and the normal group (1) after applying standardized transformation to both the male and female data sets. To evaluate odds ratios (ORs), age, blood parameters, anthropometric indices, and nutritional components were standardized. To assess for gender differences in baseline characteristics (Table 1), independent two-sample t-tests were performed.

## Results

### Association of PUD with anthropometrics, blood parameters, and nutrition in women

Tables 2 and 3 list the associations of PUD with anthropometric indices, blood parameters, and nutritional components in Korean women and men. Of all variables, age was the most strongly associated with PUD in women (p = <0.0001, OR = 0.770 [95% CI, 0.683–0.869]) and men (p = <0.0001, OR = 0.715 [0.616–0.831]).

**Table 2:** Association of peptic ulcer disease with anthropometric indices, blood parameters, and nutritional components in women.

Index	Crude		Adjustment	
	p	OR	p	OR
Age	<0.0001	0.770 (0.683–0.869)	-	-
Sleep duration	0.0222	1.153 (1.021–1.303)	0.1073	1.106 (0.978–1.251)
Pulse	0.8547	0.989 (0.876–1.116)	0.8568	0.989 (0.875–1.117)
SBP	0.2192	1.082 (0.954–1.227)	0.0003	1.309 (1.133–1.512)
DBP	0.0161	1.166 (1.029–1.321)	0.0001	1.303 (1.141–1.487)
Weight	0.0003	1.265 (1.113–1.438)	0.0008	1.251 (1.098–1.426)
Height	0.0415	1.134 (1.005–1.280)	0.8838	0.990 (0.864–1.135)
WC	0.0834	1.116 (0.986–1.264)	0.0002	1.307 (1.137–1.502)
BMI	0.0036	1.208 (1.064–1.372)	0.0001	1.303 (1.139–1.490)
WHtR	0.3022	1.067 (0.943–1.207)	0.0002	1.324 (1.144–1.532)
HipC	0.0040	1.203 (1.061–1.365)	0.0050	1.198 (1.056–1.360)
Hemoglobin	0.5681	1.036 (0.918–1.168)	0.2349	1.077 (0.953–1.217)
Platelet	0.0525	1.133 (0.999–1.285)	0.0234	1.159 (1.020–1.316)
Cholesterol	0.9954	1.000 (0.885–1.129)	0.1393	1.104 (0.968–1.259)
Triglyceride	0.4044	0.951 (0.844–1.071)	0.4637	1.050 (0.922–1.196)
HDL cholesterol	0.6790	1.026 (0.908–1.160)	0.7729	0.982 (0.867–1.112)
Glucose	0.8559	0.989 (0.878–1.114)	0.5506	1.040 (0.914–1.184)
Hemoglobin A1c	0.6177	1.039 (0.894–1.207)	0.8186	1.019 (0.867–1.198)
Energy	0.0344	1.149 (1.010–1.308)	0.2895	1.074 (0.941–1.225)
Water	0.0441	1.147 (1.004–1.310)	0.4320	1.055 (0.923–1.206)
Carbohydrate	0.0337	1.152 (1.011–1.312)	0.0979	1.117 (0.980–1.273)
Fiber	0.0065	1.212 (1.055–1.391)	0.0386	1.157 (1.008–1.328)
Ash	0.0027	1.239 (1.077–1.425)	0.0468	1.152 (1.002–1.325)
Calcium	0.0005	1.340 (1.138–1.579)	0.0079	1.243 (1.059–1.459)
Phosphorus	0.0492	1.141 (1.000–1.301)	0.4849	1.049 (0.918–1.198)
Iron	0.0414	1.166 (1.006–1.351)	0.2443	1.088 (0.944–1.254)
Sodium	0.0025	1.245 (1.080–1.434)	0.0154	1.191 (1.034–1.372)
Potassium	0.0042	1.232 (1.068–1.420)	0.0742	1.137 (0.988–1.308)
Vitamin A	0.0141	1.254 (1.047–1.503)	0.1325	1.140 (0.961–1.353)
Vitamin B2 (riboflavin)	0.0004	1.277 (1.115–1.464)	0.0477	1.155 (1.001–1.333)
Vitamin C	0.0149	1.204 (1.037–1.398)	0.1029	1.130 (0.976–1.308)
Carotene	0.0315	1.204 (1.017–1.427)	0.1820	1.115 (0.950–1.309)
Retinol	0.0889	1.455 (0.945–2.242)	0.4095	1.167 (0.808–1.686)
Niacin	0.0853	1.130 (0.983–1.299)	0.6730	1.030 (0.897–1.184)

The results of binary logistic regression, OR: odds ratio, adjustment for age, education, job, smoking, and drinking

**Table 3:** Association of peptic ulcer disease with anthropometric indices, blood parameters, and nutritional components in men.

Index	Crude		Adjustment	
	p	OR	p	OR
**Age**	<0.0001	0.715 (0.616–0.831)	-	-
**Sleep duration**	0.8476	0.985 (0.846–1.147)	0.7070	0.972 (0.840–1.126)
**Pulse**	0.1364	1.126 (0.963–1.317)	0.1477	1.123 (0.960–1.314)
**SBP**	0.0637	1.169 (0.991–1.379)	0.0042	1.276 (1.080–1.508)
**DBP**	0.0198	1.209 (1.031–1.418)	0.0212	1.208 (1.029–1.419)
**Weight**	0.0016	1.291 (1.102–1.512)	0.0881	1.159 (0.978–1.374)
**Height**	0.0740	1.147 (0.987–1.333)	0.8182	1.020 (0.864–1.204)
**WC**	0.0649	1.156 (0.991–1.348)	0.1066	1.137 (0.973–1.330)
**BMI**	0.0088	1.232 (1.054–1.440)	0.0895	1.151 (0.979–1.353)
**WHtR**	0.2493	1.094 (0.939–1.275)	0.1337	1.127 (0.964–1.319)
**HipC**	0.0003	1.326 (1.138–1.546)	0.0199	1.217 (1.031–1.435)
**Hemoglobin**	0.1484	1.112 (0.963–1.284)	0.6467	1.036 (0.891–1.205)
**Platelet**	0.9813	1.002 (0.860–1.166)	0.7822	0.979 (0.842–1.138)
**Cholesterol**	0.7270	1.028 (0.882–1.198)	0.6577	1.035 (0.888–1.207)
**Triglyceride**	0.0196	1.220 (1.032–1.441)	0.0169	1.227 (1.037–1.451)
**HDL cholesterol**	0.9142	1.008 (0.866–1.175)	0.6235	1.041 (0.887–1.221)
**Glucose**	0.1273	1.156 (0.959–1.393)	0.0452	1.220 (1.004–1.483)
**Hemoglobin A1c**	0.4044	1.548 (0.554–4.319)	0.1726	2.159 (0.714–6.522)
**Energy**	0.0688	1.165 (0.988–1.374)	0.3703	1.079 (0.913–1.275)
**Water**	0.0745	1.168 (0.985–1.384)	0.3899	1.077 (0.909–1.277)
**Carbohydrate**	0.2571	1.096 (0.935–1.285)	0.4898	1.059 (0.900–1.245)
**Fiber**	0.1069	1.146 (0.971–1.352)	0.3062	1.090 (0.924–1.287)
**Ash**	0.0484	1.190 (1.001–1.414)	0.2890	1.097 (0.924–1.302)
**Calcium**	0.0656	1.224 (0.987–1.517)	0.2328	1.131 (0.924–1.385)
**Phosphorus**	0.0109	1.253 (1.053–1.490)	0.1377	1.143 (0.958–1.363)
**Iron**	0.2213	1.115 (0.936–1.328)	0.6504	1.039 (0.879–1.229)
**Sodium**	0.0252	1.228 (1.026–1.470)	0.1005	1.160 (0.972–1.384)
**Potassium**	0.1366	1.135 (0.961–1.341)	0.6270	1.042 (0.883–1.230)
**Vitamin A**	0.6870	1.034 (0.878–1.219)	0.6362	0.963 (0.826–1.124)
**Vitamin B2 (riboflavin)**	0.1685	1.119 (0.954–1.313)	0.9771	1.002 (0.852–1.179)
**Vitamin C**	0.8809	1.012 (0.868–1.180)	0.5860	0.958 (0.821–1.118)
**Carotene**	0.8605	0.987 (0.852–1.143)	0.3341	0.933 (0.811–1.074)
**Retinol**	0.0199	1.711 (1.089–2.688)	0.1032	1.414 (0.932–2.144)
**Niacin**	0.0372	1.218 (1.012–1.465)	0.2360	1.114 (0.932–1.332)

The results of binary logistic regression, OR: odds ratio, adjustment for age, education, job, smoking, and drinking

In women, sleep duration was associated with PUD in the crude analysis (p = 0.0222, OR = 1.153 [1.021–1.303]), but the association disappeared after adjusting for age, education, job, smoking, and drinking (adjusted p = 0.1073, adjusted OR = 1.106 [0.978–1.251]). PUD was associated with diastolic blood pressure (DBP) (p = 0.0161, OR = 1.166 [1.029–1.321]) but not systolic blood pressure (SBP) (p = 0.2192, OR = 1.082 [0.954–1.227]) in the crude analysis. However, SBP and DBP were highly related to PUD after adjusting for confounders (adjusted p = 0.0003, adjusted OR = 1.309 [1.133–1.512] and adjusted p = 0.0001, adjusted OR = 1.303 [1.141–1.487], respectively).

Regarding anthropometric indices, weight was highly associated with PUD in the crude analysis (p = 0.0003, OR = 1.265 [1.113–1.438]) and remained highly associated after adjustment (adjusted p = 0.0008, adjusted OR = 1.251 [1.098–1.426]). Highly significant differences between the normal and PUD groups were found in HipC (adjusted p = 0.005, adjusted OR = 1.198 [1.056–1.360]) and BMI (adjusted p = 0.0001, adjusted OR = 1.303 [1.139–1.490]). WC and WHtR were highly associated with PUD only after adjusting for age, education, job, smoking, and drinking (adjusted p = 0.0002, adjusted OR = 1.307 [1.137–1.502] and adjusted p = 0.0002, adjusted OR = 1.324 [1.144–1.532], respectively).

After adjusting for confounders, none of the blood parameters were associated with PUD in women except for platelet count (adjusted p = 0.0234, adjusted OR = 1.159 [1.020–1.316]).

Of the nutritional components, calcium showed the strongest association with PUD in the crude analysis (p = 0.0005, OR = 1.340 [1.138–1.579]), and this association remained significant after adjusting for age, education, job, smoking, and drinking (adjusted p = 0.0079, adjusted OR = 1.243 [1.059–1.459]). Fiber and ash were significantly associated with PUD in both the crude (p = 0.0065, OR = 1.212 [1.055–1.391] and p = 0.0027, OR = 1.239 [1.077–1.425], respectively) and adjusted analyses (adjusted p = 0.0386, adjusted OR = 1.157 [1.008–1.328] and adjusted p = 0.0468, adjusted OR = 1.152 [1.002–1.325], respectively). Additionally, PUD was significantly related to sodium and vitamin B2 (riboflavin) intake in the crude analysis (p = 0.0025, OR = 1.245 [1.080–1.434] and p = 0.0004, OR = 1.277 [1.115–1.464], respectively), and these associations remained significant after adjustment (adjusted p = 0.0154, adjusted OR = 1.191 [1.034–1.372] and adjusted p = 0.0477, adjusted OR = 1.155 [1.001–1.333], respectively). Energy, water, carbohydrate, iron, potassium, vitamin A, carotene, and vitamin C were associated with PUD in the crude analysis, but these associations became non-significant after adjusting for confounders. Phosphorus, retinol, and niacin were not associated with PUD.

### Association of PUD with anthropometrics, blood parameters, and nutrition in men

DBP was significantly associated with PUD in the crude analysis (p = 0.0198, OR = 1.209 [1.031–1.418]) and after adjusting for age, education, job, smoking, and drinking (adjusted p = 0.0212, adjusted OR = 1.208 [1.029–1.419]). PUD was not associated with SBP (p = 0.0637, OR = 1.169 [0.991–1.379]) in the crude analysis but was significantly associated after adjustment for confounders (adjusted p = 0.0042, adjusted OR = 1.276 [1.080–1.508]).

Of the anthropometric indices, HipC was associated with PUD in both the crude (p = 0.0003, OR = 1.326 [1.138–1.546]) and adjusted analyses (adjusted p = 0.0199, adjusted OR = 1.217 [1.031–1.435]). PUD was related with weight and BMI in the crude analysis (p = 0.0016, OR = 1.291 [1.102–1.512] and p = 0.0088, OR = 1.232 [1.054–1.440], respectively), but these associations disappeared after adjusting for confounders.

Regarding blood parameters, triglyceride levels were associated with PUD in both the crude (p = 0.0196, OR = 1.220 [1.032–1.441]) and adjusted analyses (adjusted p = 0.0169, adjusted OR = 1.227 [1.037–1.451]); this association differed from the findings in women. Additionally, glucose was related with PUD only after adjustment (adjusted p = 0.0452, adjusted OR = 1.220 [1.004–1.483]).

Finally, for the nutritional components, PUD was associated with ash (p = 0.0484, OR = 1.190 [1.001–1.414]), phosphorus (p = 0.0109, OR = 1.253 [1.053–1.490]), and sodium (p = 0.0252, OR = 1.228 [1.026–1.470]) in the crude analysis but not after adjustment for confounders. Additionally, retinol and niacin were related to PUD (p = 0.0199, OR = 1.711 [1.089–2.688] and p = 0.0372, OR = 1.218 [1.012–1.465], respectively) in the crude analysis, but these associations disappeared after adjustment.

## Discussion

PUD is a major condition affecting the digestive system [1, 2]. In the present study, we identified the associations of PUD with anthropometric indices, blood parameters, and nutritional components in Korean men and women.

Anthropometric indices have been used in studies assessing various diseases including hypertension, diabetes, hypertriglyceridemia, hyper-LDL-cholesterolemia and hypo-HDL-cholesterolemia, dyslipidemia, and albuminuria [42–44]. Previous studies have examined the association of PUD with anthropometric indices such as BMI, WC, and WHtR. However, these associations remain controversial. Several studies have reported that PUD is associated with anthropometric indices [15–17], whereas others have suggested that they are not related [2, 18, 19]. Recently, Boylan and colleagues [16] examined the association of PUD with BMI and WHtR in a prospective cohort study called the Health Professionals Follow-Up Study, and they found that BMI and WHtR were risk factors of PUD. Additionally, Kalichman and colleagues [15] evaluated the association of PUD, diabetes, hypertension, and ischemic heart disease with anthropometric indices in Russian men and women, and they showed that PUD and gastritis were strongly associated with BMI, WC, and WHtR. Weatherall and colleague [17] reported that BMI was positively associated with ischemic heart disease, diabetes mellitus, and blood pressure in middle-aged British men and that PUD was inversely associated with BMI. However, Tsai and colleagues [18] observed a non-significant effect of BMI on PUD in Taiwanese men and women. Rosmond and colleagues [19] examined that relationship between PUD and anthropometric indices in Swedish men and argued that PUD was not associated with BMI or WHtR. Furthermore, Cheng and colleagues [2] did not find an association between PUD and BMI in US adults. Our findings are consistent with the results of previous studies [15–17]; specifically, we found that weight, HipC, and BMI in women and HipC in men were highly associated with PUD in both the crude and adjusted analyses. Therefore, we suggest that PUD may be associated with anthropometric indices such as weight, BMI, and HipC in women, but not in men, although there was not a strong association between PUD and anthropometric indices in the Korean population.

Regarding nutritional components, some studies have argued that high dietary fiber intake is associated with PUD, gastric cancer, and gastroesophageal reflux disease, when compared with low dietary fiber intake [20–23, 45], and that intake of vitamin A and C is related to PUD [21, 22, 25]. Anderson and colleagues [20] suggested that a high intake of dietary fiber reduced the risk of gastrointestinal and duodenal ulcer, coronary heart disease, diabetes, obesity, and stroke. Additionally, Ryan-Harshman and Aldoori [21] documented that a high-fiber diet and soluble fiber intake appeared to decrease the risk of duodenal ulcer. Aldoori and colleagues [22] reported that dietary fiber intake was inversely related to the risk of duodenal ulcer in US men when comparing the highest and lowest quintiles of dietary fiber intake. Furthermore, they demonstrated that soluble fiber was highly associated with a reduced risk of duodenal ulcer. The findings of previous studies are consistent with the results of our study in women, but not in men. In the present study, fiber was associated with PUD in women in the crude and adjusted analyses, but it was not associated with PUD in men. In another study, Aldoori and colleagues [22] found that vitamins A and E were associated with the risk of duodenal ulcer and that vitamin A and fiber could possibly reduce the development of duodenal ulcer. They argued that vitamin B2 and potassium were inversely associated with the risk of duodenal ulcer. Miyake and colleagues [24] reported that a lack of water-soluble vitamins could accelerate the development of PUD in Japanese adults. Additionally, Aditi and Graham [25] documented that Vitamin C (ascorbic acid) plays a very important role in the conservation and treatment of the gastric mucosa and that deficiency in vitamin C has repeatedly been linked with PUD. Our findings in women are consistent with the results of previous studies [22, 24, 25]. In this study, fiber, vitamin B2 (riboflavin), sodium, and ash were significantly associated with PUD in women in both the crude and adjusted analyses. However, in men, our results do not agree with previous findings. Interestingly, our analysis of nutritional components showed that calcium had the strongest association with PUD in women in both the crude and adjusted analyses.

In a previous study, Kuri-Morales and colleagues [26] examined the prevalence of stroke, diabetes, and PUD according to age in both Mexican men and women. They showed that the prevalence of PUD in men and women increased with increasing age. Additionally, Del Vecchio Blanco [46] reported that PUD increased with age in Italian men and women. Sonnenberg and colleagues [3] reported that the occurrence of duodenal ulcer and gastric ulcer was the same in adult US men and women and that age, smoking, and education were associated with PUD. However, Cheng and colleagues [2] assessed the association of physical activity with the incidence of PUD in US men and women and could not find an association of age with any type of ulcer disease. The findings of our study are consistent with the results of previous studies [3, 26, 46]; of all the variables assessed in this study, age was the most strongly associated with PUD in both women and men.

Ko and colleagues [28] assessed the association between sleep duration and PUD in Korean men and women and reported that women who slept for more than 9 hours were significantly less likely to have PUD than those who slept 7 hours; additionally, men with a sleep duration of more than 9 hours tended to have a reduced risk of PUD. The results of this previous study [28] do not agree with our findings. In our study, although sleep duration was associated with PUD in the crude analysis, the association was not significant after adjusting for age, education, job, smoking, and drinking. Additionally, in men, sleep duration was not associated with PUD in either the crude or adjusted analyses.

Regarding the association between blood pressure and PUD, Segawa and colleagues [47] reported that DBP differed significantly between the PUD group and normal group in Japanese women and that SBP and DBP were inversely associated with the incidence of gastric and duodenal ulcer in men. By contrast, Sonnenberg [48] argued that duodenal ulcer was not a risk factor for diseases associated with hypertension in German. We found that DBP in women and men was associated with PUD in the crude and adjusted analyses, whereas SBP in women and men was associated with PUD in the adjusted analysis only.

Finally, our findings regarding blood parameters differed between women and men. None of the blood parameters was associated with PUD in women in the crude and adjusted analyses, whereas triglycerides were associated with PUD in men in both the crude and adjusted analyses.

The present study has several limitations. First, this study used a cross-sectional design, and therefore the causal relationships between PUD and the studied variables could not be ascertained. Second, the results may have been affected by a number of biases including recall bias, survey bias, sampling bias and confounding. Third, our findings may not be consistent with results from other countries because characteristics such as dietary behaviors and the prevalence of PUD vary among different locations. Finally, we focused on PUD criteria including gastritis, gastric ulcer, and duodenal ulcer in this study. Therefore, the limitations of this study include the relatively small sample size and short study duration because the analysis is based only on the KNHANES I data set. As a next step in our research, we will explore the differences in nutritional components, lifestyles, and anthropometric indices according to different diagnostic criteria of KNHANES I and KNHANES II-VI by using a large-scale representative data set (KNHANES II-VI). Despite these limitations, to our knowledge, this is the first national study on the associations of PUD with anthropometric indices, blood parameters, and nutrition in a representative Korean population.

## Conclusions

In the present study, we evaluated the association of PUD with anthropometrics, blood parameters, lifestyle, and nutrition in the Korean population. Our findings suggest that age is the factor most strongly associated with PUD in Korean adults, that obesity is associated with PUD, that the association between nutritional components and PUD is greater in women than in men, and that there are few relationships between blood parameters and PUD. In summary, our main findings are as follows. 1) Increased age was a strong risk factor for PUD in both Korean women and men. 2) In anthropometric indices related to obesity, decreased weight, BMI, and HipC values corresponded to an elevated risk of PUD in Korean women, and decreased HipC values corresponded to an increased risk of PUD in Korean men. 3) Regarding nutritional components, increased intake of dietary calcium decreased the risk of PUD in women, but not in men. Additionally, increased intake of dietary fiber, ash, sodium, and vitamin B2 decreased the risk of PUD in women. These results indicate that anthropometrics, lifestyle, and nutrition could play a significant role in PUD in Korean adults. Furthermore, the findings provide useful information for PUD screening tools and could improve public health efforts to address PUD.

## Supporting information

S1 Fig Sample selection procedure used in this study. (PDF)
