# Supplementary figures and images for: Association of peptic ulcer disease with obesity, nutritional components, and blood parameters in the Korean population
Source: PLoS One. 2017 Aug 24;12(8):e0183777. doi: 10.1371/journal.pone.0183777 (PMC5570349; doi:10.1371/journal.pone.0183777)

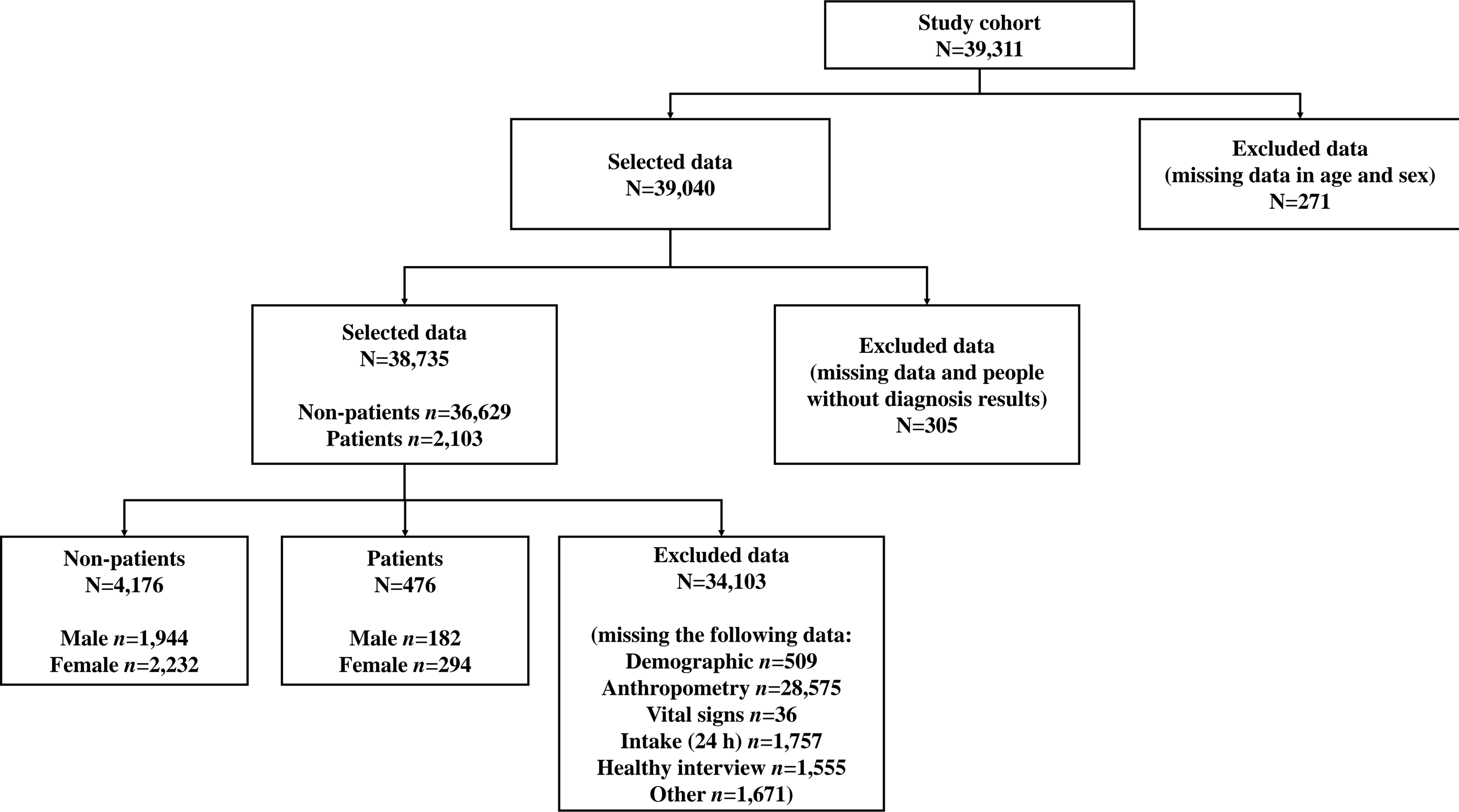

Supplement: S1 Fig — (PDF) [file pone.0183777.s001.pdf]
